# The Deathly Hallows of the Austrian Triad

**DOI:** 10.7759/cureus.6568

**Published:** 2020-01-05

**Authors:** Abeera Akram, Ahmed Kazi, Abdul Haseeb

**Affiliations:** 1 Internal Medicine, Saint Mary's Hospital, Waterbury, USA

**Keywords:** endocarditis, pneumonia, meningitis, infectious, cardio-embolic stroke, steroids

## Abstract

We have a case vignette of a 67-year-old gentleman who presented in with altered mentation and sepsis. During his hospital course, he was diagnosed with meningitis, endocarditis, and pneumonia hence completing the Austrian triad. He had no identifiable risk factors, but because of the timely diagnosis, he was given optimum treatment. He improved clinically and was discharged to a rehabilitation facility. Austrian syndrome is a pathological diagnosis caused by disseminated Streptococcus pneumoniae infection, characterized by the triad of pneumonia, endocarditis, and meningitis. We present this successfully treated case of a patient with no identifiable risk factors presenting as disseminated streptococcal pneumoniae infection.

## Introduction

Osler’s Triad (Austrian syndrome) is a rare but deadly triad comprising meningitis, endocarditis, and pneumonia. It is a disseminated infection caused by Streptococcus pneumoniae. It is an encapsulated gram-positive coccus that resides in the human respiratory tract. It carries higher mortality and morbidity rates despite aggressive treatment. Since the addition of a 13-valent pneumococcal conjugated vaccine to the list of routine childhood immunizations, the epidemiology of this triad has changed [1]. We present a successfully treated case of Austrian syndrome in a 67-year-old gentleman who presented with altered mentation and was admitted to a critical care unit. With the timely diagnosis and optimized treatment, he was successfully treated for this infection, which if left untreated can attack various organs in the body. We also present a literature review of several case reports discussing this triad and its proper treatment [1-5].

## Case presentation

This is a 67-year-old gentleman with no known past medical history, who presented in the Emergency Department (ED) with aphasia and confusion. History was obtained from the patient's girlfriend who revealed that he had been confused for the last one week but on the day prior to his presentation there was an increase in confusion along with an inability to speak, move his extremities or respond to commands. Vital signs were significant for a temperature of 100.5° F. He was hypoxic requiring 4 liters of oxygen via nasal cannula to maintain a saturation above 92%. Pertinent physical examination findings included a grade 2 systolic murmur in aortic area, coarse bibasilar crackles, extended up to mid thorax and a Stroke scale score of 24 (Table 1).

**Table 1:** The National Institutes of Health Stroke Scale.

Stroke Scale	
Level of consciousness (LOC)	1
LOC Questions	2
LOC Commands	1
Visual	0
Facial palsy	1
Motor Arm, Left	1
Motor Arm, Right	1
Motor Leg, Left	3
Motor Leg, Right	3
Limb Ataxia	2
Sensory	2
Best Language	3
Dysarthria	2
Extinction and Inattention	2
Total	24

**Figure 1:**
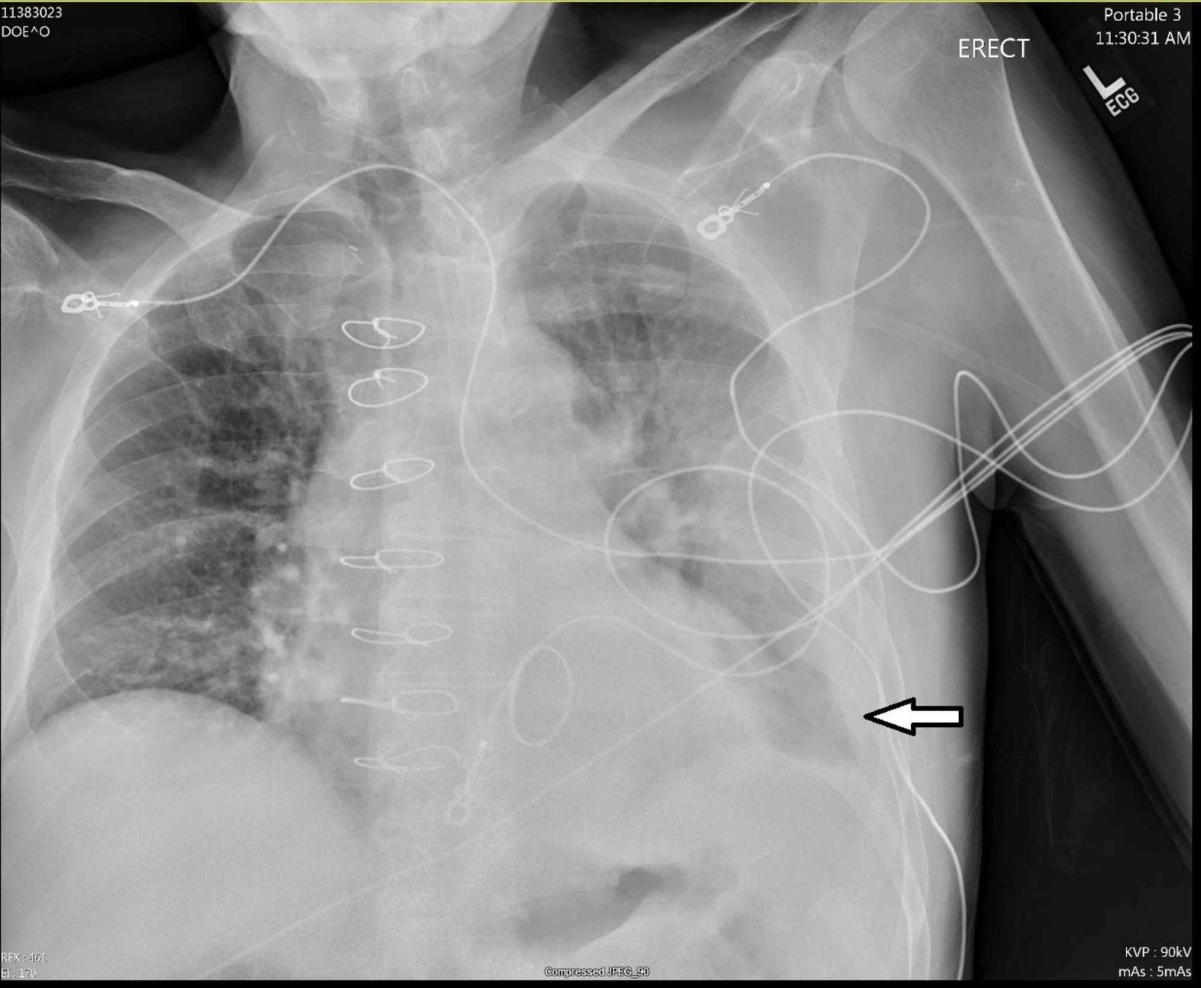
Chest X-Ray. Arrow pointing towards area of consolidation on left basilar region.

Lab work was significant for a white blood cell count of 17.7 (4.0-10.5 k/uL), potassium 3.4 (3.5-5.1 mEq/L), and a brain-natriuretic peptide level of 1006 (0.0-100.0 pg/mL). Chest X-ray demonstrated mild cardiomegaly status post cardiac valve replacement, moderate left pleural effusion, left peri-hilar and basilar consolidation (Figure 1).

**Figure 2:**
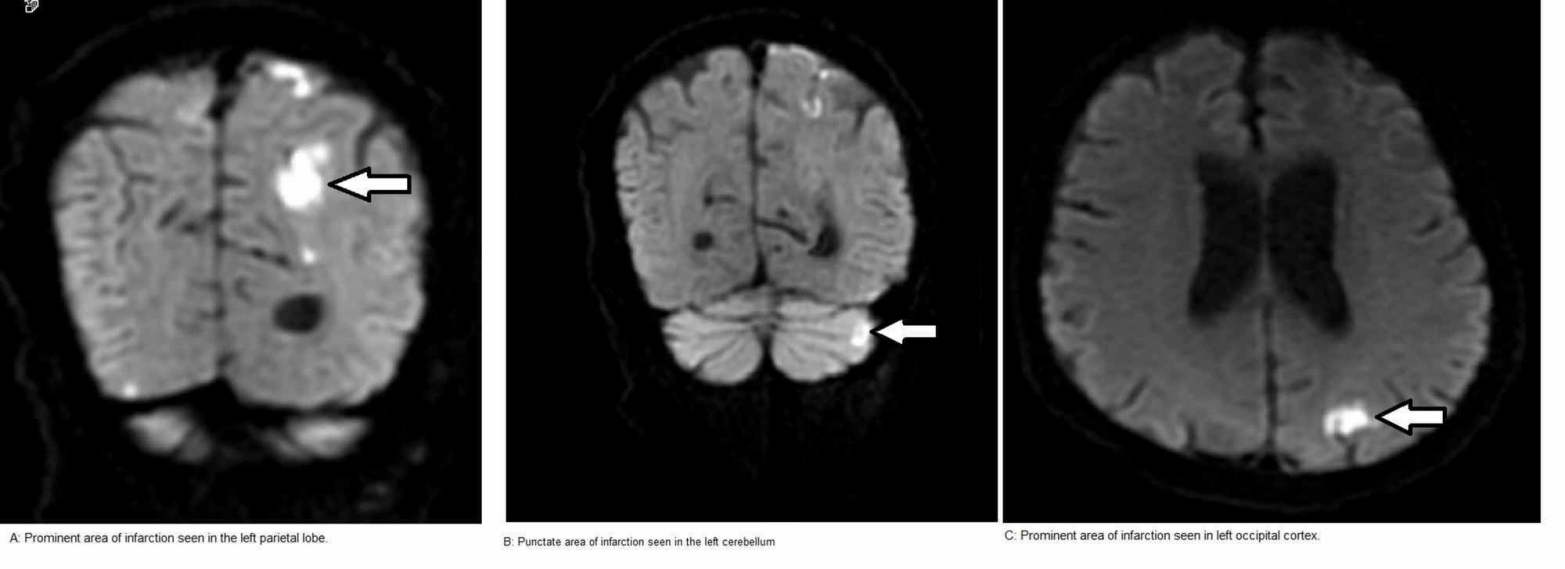
MRI of the head. Arrows showing multiple areas of infarct in left parietal (A) and occipital (C) cortex and left cerebellum (B).

Stroke alert was called and a stat CT head was obtained which did not show any infarct. The neurologist deferred intra-venous (IV) tissue plasminogen activator or thrombectomy as it was unclear how long the patient had been symptomatic. Later an MRI of the head was obtained, which showed a prominent area of an acute infarction in the left posterior parietal lobe. In addition, there were punctate areas of acute infarction in the left parietal subcortical region, left sub-insular region, right parietal cortex, right occipital cortex and left cerebellum (Figure 2).

Findings were suggestive of multiple embolic infarcts.

The patient was initially admitted to a step-down telemetry unit with a concern of sepsis secondary to pneumonia. He was started on empiric antibiotics. Meningoencephalitis was also one of concern, so a lumbar puncture (LP) was done. The patient's condition was deteriorating, so he was transferred to the Intensive Care Unit (ICU) for further care. His cerebral spinal fluid (CSF) results showed low glucose, high proteins, only 63 leukocytes but cultures did not show any growth (there was a glitch that antibiotics were administered before getting LP).

While in the ICU, antibiotics were continued and he was started on dexamethasone 0.15 mg/kg for concern of pneumococcal meningitis. The following day, the blood cultures, previously obtained, revealed 2 of 2 sets positive for streptococcus pneumoniae. An electroencephalogram (EEG) was performed, which was abnormal, a seizure focus was not reliably ruled out but the patient was started on antiepileptic with Valproic acid.

Thoracentesis was also attempted to drain the pleural effusion but was unsuccessful due to scant pleural fluid. Infectious disease was consulted, who suggested that a patient could possibly have an Austrian syndrome as he had pneumonia, meningitis and possible endocarditis (given multiple embolic infarcts).

Later, trans-thoracic echo (TTE) was obtained, which did not rule out vegetation, hence a trans-esophageal echo (TEE) was performed, which showed small mobile vegetation seen on the mitral valve anterior leaflet with severe mitral regurgitation. With these findings, the patient met the Dukes criteria for infective endocarditis. He was also seen by a cardio-thoracic surgeon who recommended no surgical intervention.

As it was proven that he had pneumonia, infective endocarditis, and possible meningeal encephalitis, he was met the triad of Austrian syndrome. Antibiotics were narrowed only to penicillin according to susceptibility. With this change in care, the patient started to improve clinically within 24 hours. After the fourth day of his hospitalization, he was discharged to an acute rehab center with instructions to continue the present antibiotic six weeks.

## Discussion

The deadly triad of Austrian syndrome comprises meningitis, infective endocarditis, and pneumonia caused by Streptococcus pneumoniae (S. pneumoniae) dissemination. This clinical triad has an incidence of 0.9-7.8 cases per 10 million people each year with a mortality of about 32% [1]. Before the discovery of penicillin in the 1940s, S. pneumoniae was responsible for 15-20% of all endocarditis cases, but this number has decreased presently to less than 3% now [2,3]. It is also believed that the advent of the 13-valent pneumococcal conjugated vaccine in children has led to a further decrease in the incidence of Austrian syndrome.

Usually, this syndrome is found in middle-aged alcoholic men [1]. The precise mechanism of how alcoholism precipitates to this lethal infection is not well understood but may be due to hyposplenism, defective leukocyte response, and chemotaxis [3]. Other risk factors include cirrhosis, splenectomy, and immunodeficiency. The aortic valve is the most common site of endocarditis in this condition. Medline search was performed in 2008. It revealed that aortic insufficiency was the commonest reason for cardiac failure, ultimately requiring valve replacement [6]. This was not the case in our patient. He had mitral valve involvement for which he underwent valve replacement earlier in his life (not native).

The most common complication of this syndrome is congestive heart failure (CHF). Other, less commonly seen, suppurative complications include aortic abscesses, septic brain emboli, arthritis, aneurysm, and septic arthritis [3].

Timely diagnosis and appropriate antimicrobial therapy, and, if indicated, early surgical intervention of the involved cardiac valve decreases morbidity and mortality. Early recognition of the disease is easily missed as the clinical features overlap with various differentials such as stroke, encephalopathy, congestive heart failure, etc. It complicates management. Empirically, disseminated S. pneumoniae infection is treated with vancomycin with ceftriaxone or cefotaxime. Antibiotics can be narrowed down based on the results of in vitro susceptibility testing, as we did in our patients. Vancomycin should be continued if a high-level penicillin resistance has been documented [7]. Also, a study done by de Gans and van de Beek showed early treatment with dexamethasone in acute pneumococcal meningitis improves the outcome as compared to placebo, a reason why we started our patient on dexamethasone [8].

In another case report, a 44-year-old patient with a history of insulin-dependent diabetes mellitus presented to the emergency department with altered mental status, high fever, and meningeal signs. The patient was found to have Austrian syndrome. He was treated with empiric antibiotics and survived the captain of the dead but his quality of life was impaired by the post-recovery sequelae, including dyspnea, cognitive impairment and hearing loss [1]. There was one case report found involving a 76-year-old woman who presented with a three-day history of headache and was ultimately found to have Austrian syndrome. Authors claimed her to be the oldest patient in the reviewed literature for Austrian syndrome [2]. A 61-year-old man presented with fever and altered mental status fulfilled the triad of this syndrome, ultimately required aortic valve replacement before being discharged [3]. Another case report describes a Bangladeshi 48-year-old man who was diagnosed with Austrian syndrome and successfully treated by medical management [4]. We also found an interesting case in the context of a fulminant pneumococcal native valve endocarditis. He was a 41-year-old male, who presented in cardiogenic shock and was ultimately found to have massive native valve endocarditis along with pneumonia and meningitis. He had cardiac surgery, which revealed extensive involvement of the aortic root, aortic valve, mitral valve and destruction of aortomitral continuity. Unfortunately, the patient died intraoperatively while ultima ratio procedure surgery was being done [5].

Our case has a few exciting teaching points. Initially, the change in the mental status of our patient was very vague and was attributed to strokes. Initially, he was just confused, but when he developed aphasia, he was finally brought to the hospital. Also, since the patient was not an IV drug user, the diagnosis of pneumococcal endocarditis was not considered until later in the hospital course, aided by the positive blood cultures. One good decision which the treatment team took was to transfer the patient to the intensive care unit for closer monitoring. This resulted in prompt testing and treatment. Through our case, we wish to illustrate the importance of maintaining a high degree of suspicion for Austrian syndrome in all patients with severe sepsis and suspected pneumococcal infection, even if they do not have predisposing risk factors.

## Conclusions

This concomitant infection involving all three organs (brain, lungs, and heart) known as Austrian syndrome is seen infrequently in this antibiotic era but is still associated with a poor outcome. Hence early recognition and appropriate medical or combined medical-surgical treatment need to be considered promptly.
